# Supplementary figures and images for: The Human ApoE4 Variant Reduces Functional Recovery and Neuronal Sprouting After Incomplete Spinal Cord Injury in Male Mice
Source: Front Cell Neurosci. 2021 Feb 18;15:626192. doi: 10.3389/fncel.2021.626192 (PMC7930340; doi:10.3389/fncel.2021.626192)

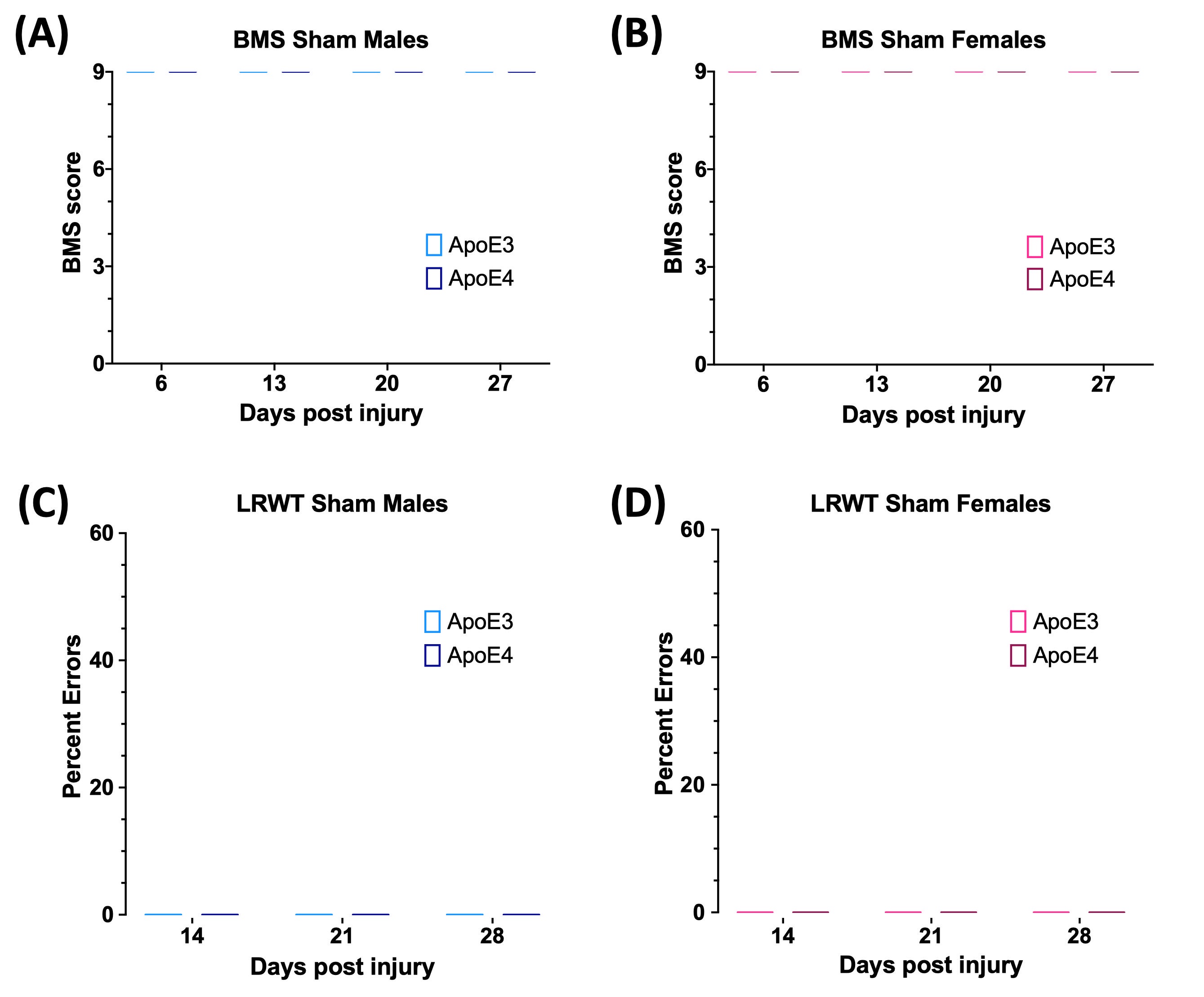

Supplement: Supplementary Figure 3 — Behavioral test results in ApoE3 and ApoE4 Sham mice. Weekly BMS evaluation for ApoE3 and ApoE4 male (A) and female (B) mice starting at 6 and up to 27 days after laminectomy-only surgeries. Weekly LRWT starting at 14 days for ApoE3 and ApoE4 male (C) and female (D) sham mice. BMS scores range between 0 and 9; LRWT is expressed as percent of foot placement errors. Box-and-whisker diagrams depicting the data are presented. No statistical differences were observed. N = 10 for male and female shams. [file Image_3.JPEG]
